# Effect of metabolic syndrome components on the risk of malignancy in patients with gallbladder lesions

**DOI:** 10.7150/jca.54617

**Published:** 2021-01-01

**Authors:** Zheng Deng, Yan Xuan, Xinxing Li, William J Crawford, Zhiqing Yuan, Zhoukan Chen, Anastasia Brooks, Yanyan Song, Haolu Wang, Xiaowen Liang, Tao Chen

**Affiliations:** 1 Department of General Surgery, South Campus, Renji Hospital, School of Medicine, Shanghai Jiaotong University, Shanghai, 201112, China; 2 Department of Endocrinology, Luwan Branch, Ruijin Hospital, School of Medicine, Shanghai Jiaotong University, Shanghai, 200020, China; 3 Department of Gastroenterological Surgery, Tongji Hospital, School of Medicine, Tongji University, Shanghai, 200065, China; 4 Department of General Surgery, Changzheng Hospital, The Second Military Medical University, Shanghai, 200003, China; 5 Department of Medical Sciences, University of Oxford, Oxford OX3 9DU, United Kingdom; 6 Department of General Surgery, Luwan Branch, Ruijin Hospital, School of Medicine, Shanghai Jiaotong University, Shanghai, 200020, China; 7 The University of Queensland Diamantina Institute, The University of Queensland, Brisbane, QLD, 4102, Australia; 8 Gallipoli Medical Research Institute, Greenslopes Private Hospital, Brisbane, QLD, 4120, Australia; 9 Department of biostatistics, clinical research institute, School of Medine, Shanghai Jiaotong University, Shanghai, 200025, China; 10 Department of Biliary-Pancreatic Surgery, Renji Hospital, School of Medicine, Shanghai Jiao Tong University, Shanghai, 200127, China; Zheng Deng, Yan Xuan and Xinxing Li contributed equally to this work.

**Keywords:** Gallbladder lesions, Benign gallbladder polyps, Gallbladder cancer, Metabolic syndrome components, Dyslipidemia, High density lipoprotein

## Abstract

**Background:** Gallbladder lesions have become more common nowadays. But there is limited evidence-based guidance on surveillance of these patients for malignancy. Predicting malignancy could help clinicians better manage this condition and improve the prognosis. We evaluated the independent and joint effects of metabolic syndrome components on the risk of malignancy among patients with gallbladder lesions.

**Methods:** Using a multicenter database, consecutive patients with pathologically confirmed gallbladder lesions between 2012 and 2019 were identified. Univariate and multivariate logistic regression analyses were used to evaluate the effects of metabolic syndrome components (diabetes, hypertension, dyslipidemia and obesity) as additive or combined indicators for the risk of malignancy. Unadjusted and adjusted odds ratios were calculated.

**Results:** Of the 625 patients, 567 patients were identified with benign gallbladder lesions and 58 patients with gallbladder cancer (GBC). GBC group had less obesity but more dyslipidemia. Among all metabolic syndrome components, only dyslipidemia was significantly associated with GBC (odds ratio 2.674, 95% confidence interval 1.173-6.094). Dyslipidemia was an independent risk factor for malignancy (adjusted odds ratio 2.164, 95% confidence interval 1.165-4.021), regardless of whether the other risk factors and metabolic syndrome components were combined. Patients with decreased high-density lipoprotein had 3.035-fold higher risk of malignancy (adjusted odds ratio 3.035, 95% confidence interval 1.645-5.600).

**Conclusions:** Dyslipidemia is associated with a 2.674-fold increase in the risk of malignancy in patients with gallbladder lesions. Dyslipidemia is an independent risk factor for malignancy, regardless of the presence of the other risk factors and metabolic syndrome components.

## Introduction

Gallbladder lesions have become more common nowadays with the increased use of ultrasound 1. The incidence rate of gallbladder lesions is approximately 5-9.9% of the population 2. Cholesterol gallbladder polyps are the most common type of benign gallbladder polyps (BGP). Some BGP, such as adenomas, are considered with malignant potential 1. About 3-8% of all gallbladder lesions are malignant 3. Current European consensus guideline suggests that all gallbladder lesions greater than 10 mm should be surgically removed, and those between 6 and 9 mm with co-existing presence of high risk factors are deemed to justify cholecystectomy 4. However, there is limited evidence-based guidance on how to manage patients who were not offered surgical treatment 5. It has been suggested patients with gallbladder lesions less than 10 mm could be followed conservatively 1, 4. For such surveillance to be cost-effective, better risk stratification is needed to guide targeted surveillance. Predicting malignancy by better understanding risk factors would also allow clinicians to more effectively plan secondary prevention efforts of gallbladder cancer (GBC) for patients with gallbladder lesions 6, 7.

The metabolic syndrome components (diabetes, hypertension, dyslipidemia and obesity) are readily identifiable and potentially modifiable, rendering them as ideal targets not only for risk stratification but also for risk modification, which would be useful to both predicting prognosis and preventing complications. Some manifestations of metabolic syndrome (such as diabetes, hyperlipidemia and obesity) are the risk factors for gallbladder stones or lesions 8. Diabetes was associated with biliary tract cancers 9.

However, until now, it is still controversial whether the metabolic syndrome components could be involved in the malignant risk scoring systems for gallbladder lesions. Available studies followed only a limited number of patients with incomplete risk factor data 10, 11. There remains doubt about the association between metabolic syndrome components and the risk of biliary tract cancers 12. The frequent interaction and co-occurrence of these metabolic syndrome components also complicates the analysis of each component's specific contribution to the risk of malignancy in patients with gallbladder lesions.

To fill this gap, we conducted a retrospective cohort study of 625 patients with pathologically confirmed gallbladder lesions from three hospitals in China. The independent and joint effects of syndrome components on the risk of malignancy were evaluated.

## Method

### Study population and laboratory tests

Using a multicenter database, a total of 625 consecutive postoperative patients who pathologically diagnosed with gallbladder lesions from February 2012 and December 2019 at three Chinese hospitals (Renji Hospital, Ruijin Hospital and Shanghai Changzheng Hospital) were identified. A standardised data form was created to collect all relevant information including age, gender, body mass index, hypertension, diabetes, the data of ultrasonography including number of gallbladder lesions (single or multiple), accompanying with stones and maximum diameter of lesions and laboratory findings including fasting blood glucose, liver function tests and lipid profiles. The inclusion criteria for the patients with gallbladder lesions were as follows: (a) patients underwent surgical treatment and had pathologically confirmed diagnosis; (b) examination with ultrasonography was performed within 1 month before surgery. We excluded patients diagnosed with other cancers within the five years before the diagnosis of gallbladder lesions to ensure metastatic cancers were excluded. Patients younger than 18 years or with missing any of the required data were excluded. Dyslipidemia was defined when any of the following criteria were satisfied: 1) hypercholesterolemia: ≥ 6.2 mmol/L; 2) hypertriglyceridemia: ≥ 2.3 mmol/L (200 mg/dL); 3) low high density lipoprotein (HDL) cholesterol: <1.03 mmol/L (40 mg/dL) in men and <1.29 mmol/L (50 mg/dL) in women. Obese was identified by BMI ≥ 25kg m^-2^ according to the WHO classification for Asian populations. The study was conducted in accordance with the Declaration of Helsinki and was approved by the Institutional Ethical Review Committee of Renji Hospital (Ethical Approval Number 2016-045).

### Histopathology

All gallbladder lesions underwent an independent review by two pathologists. In this study, BGP include cholesterol polyps, inflammatory polyps, adenoma, adenomyomatosis, cholesterolosis and hyperplasia. GBC only include adenocarcinoma 13.

### Statistical analysis

Data on continuous variables were expressed as mean ± standard deviation (SD), and the categorical variables are summarised as frequencies and percentages. T tests were conducted for continuous variables and χ2 tests for categorical variables. Odds ratios (OR), 95% confidence interval (CI) and *P* values were calculated for each variable. The multivariate logistic regression models were constructed to examine the risk of malignancy. The univariate and multivariate models were constructed to test the joint association between dyslipidemia and the other metabolic syndrome components and restricted one risk factor without the others. Models were adjusted for age and gender. The results were considered statistically significant when *P* values were < 0.05. The adjusted ORs and 95% CIs were calculated for each parameter estimate. All statistical analyses were performed by SPSS version 25 (IBM Co., Armonk, NY, USA).

## Results

### Patient characteristics

We identified 625 patients with gallbladder lesions in total. As shown in Table 1, 567 patients had BGP and 58 had GBC. There was no significant difference in gender between the two groups. The mean age of the two groups was 49.94 ± 12.73 and 65.84 ± 9.98 years old, respectively. Patients in GBC group were significantly older than those in BGP group (*P* < 0.001) or the gallbladder adenomatous polyp group (*P* < 0.001, Supplementary Table 1). Multiple lesions were found more frequently in patients with BGP than in those with GBC (64.6% vs 29.3%, *P* < 0.001). Comparing to the BGP group, the level of total bilirubin was significantly higher in GBC group (*P* = 0.001, Table 1) or adenomatous polyp group (*P* = 0.009, Supplementary Table 1). Among all patients, the BGP, adenomatous polyp and GBC had different mean lesion diameters of 6.49 ± 4.60 mm, 9.27 ± 5.70 mm and 26.26 ± 22.66 mm, respectively (*P* < 0.001). Accompanying gallstones was more common in the GBC group (3.4%) than the BGP group (20.8%, *P* = 0.001) or the adenomatous polyp group (25.4%, *P* = 0.001, Supplementary Table 1).

Obesity was more common in BGP group compared to GBC group (Table 1, 35.6% vs 22.4%, *P* = 0.044). Dyslipidemia was more common in GBC group than BGP group (63.8% vs 42.2%, P = 0.002). Decreased HDL was more common in the GBC group than BGP or adenomatous polyp groups (*P* < 0.001, Supplementary Table 1). There was no significant difference in total cholesterol (15.3% vs 10.3%), triglycerides (TG, 12.7% vs 15.5%), hypertension (48.5% vs 58.6) and diabetes (11.3% vs 17.2%) between the BGP and GBC groups.

### Independent risk factors for GBC

Univariate and multivariable analyses were performed to identify independent risk factors for GBC (Table 2). A single factor regression analysis on all indicators revealed that age, number of polyps, total bilirubin, maximum diameter of polyps, accompanying stones, BMI and dyslipidemia were associated with an increased risk of GBC. Dyslipidemia was associated with a 2.4-fold increase in the odds of GBC (*P* = 0.002).

These risk factors were then included in the multivariate analysis, which was adjusted for age and gender. As shown in Table 2, dyslipidemia and size of polyps were associated with a 2.6-fold and 1.162-fold increase in risk of malignancy in patients with gallbladder lesions. For patients with neoplastic gallbladder lesions, decreased HDL was associated with a 5-fold increase in risk of malignancy in (95% CI: 1.502-16.801, *P* = 0.009, Supplementary Table 2).

### Joint associations of metabolic syndrome components with GBC

Metabolic syndrome components often coexist in patients. Then we examined whether a specific combination of them was associated with an increased risk of GBC. Table 3 summarised the different types of metabolic characteristics in the two groups. Six combinations of metabolic conditions were more common in GBC group than BGP group, including diabetes without BMI ≥ 25, dyslipidemia without BMI ≥ 25, hypertension without BMI ≥ 25, diabetes without hypertension, dyslipidemia and hypertension, and dyslipidemia and diabetes.

To verify whether these factors increased the risk of malignancy, both univariate and multivariate analyses were performed (Table 4). In the univariate analysis, we found that hypertension without BMI ≥ 25 (OR=1.910, 95% CI: 1.097-3.325), with dyslipidemia without BMI ≥ 25 (OR=2.857, 95% CI: 1.652-4.924), with both dyslipidemia and hypertension (OR=2.548, 95% CI: 1.456-4.458) and with both dyslipidemia and diabetes (OR=3.329, 95% CI: 1.432-7.740) were associated with risk of malignancy in patients with gallbladder lesions. The multivariate analysis model was adjusted for age and gender, and dyslipidemia excluding diabetes group and dyslipidemia excluding obese group were significantly associated with GBC. Dyslipidemia without diabetes, and dyslipidemia without BMI ≥ 25 were both associated with an approximately 1.9- and 2.5-fold increase in the risk of malignancy in patients with gallbladder lesions. The trait of hypertension without dyslipidemia seem to be a protective factor for patients (OR=0.438, 95% CI: 0.201-0.953). There was no other significant interaction among dyslipidemia and other risk factors. These results indicated that dyslipidemia is an independent risk factor for increase in the risk of malignancy, regardless of the presence of other metabolic syndrome components.

### Analysis of dyslipidemia types for GBC

Our results show that dyslipidemia is a risk factor for GBC. We then examined this association with different dyslipidemia types, including hypercholesterolemia, TG, increased low density lipoprotein (LDL) and decreased high density lipoproteins (HDL). In both univariate and multivariable analyses, dyslipidemia and decreased HDL were closely associated with increased the risk of malignancy in patients with gallbladder lesions (Table 5) or with neoplastic gallbladder lesions (Supplementary Table 2), while elevated total cholesterol level or TG level had no significantly impact on the risk of malignancy.

## Discussion

Although the incidence of GBC is low, its prognosis is extremely poor 14, 15 with a 5-year survival rate less than 20% 16. Predicting the risk of malignancy of gallbladder lesions could help clinicians manage this condition and potentially improve the prognosis 17. A recent systematic review revealed the role of diabetes in gallbladder diseases 18. However, the specific individual diabetic or other metabolic risk factors are still unclear. This is the first study that elucidated the association between detailed metabolic syndrome components (diabetes, hypertension, obese and dyslipidemia) and the risk of malignancy in patients with gallbladder lesions.

We found that the GBC group had older age, larger size of polyps, more common presence of gallbladder stones, obesity and dyslipidemia compared with the BGP group. In the multivariate analysis, single polyp and elevated bilirubin level had no significant association with malignancy, but dyslipidemia was a significant risk factor for GBC. The older age and larger polyp diameter have been reported as risk factors of GBC 10, 19, 20, which were similar to our results.

In this study, dyslipidemia was identified as an independent risk factor for malignancy, regardless of the presence of diabetes, hypertension and obese. Dyslipidemia was present in 44% of patients with gallbladder lesions and associated with a 2.6-fold increased risk of malignancy. These findings suggest that dyslipidemia may contribute to the malignancy progression of gallbladder lesions. Elevated total cholesterol, elevated TG and decreased HDL have been associated with an 18%, 15%, and 20% increased risk of cancer, respectively 21. Previous studies also indicated that statin, a cholesterol-lowering medication, could decrease the risk of developing cancers 22. Dyslipidemia also closely related to oxidative stress. Dyslipidemia downregulated the HDL antioxidant/ anti-inflammatory function and increased oxidative stress and inflammatory, which have been proven to be contributing factors for cancers 23.

Individual metabolic syndrome components are closely related and frequently co-occur. Dyslipidemia is usually a feature in patients with type 2 diabetes and obesity. Patients with obesity and diabetes frequently exhibit elevated circulating cholesterol, TG, and reduced HDL 24. Diabetes, obesity and dyslipidemia contribute to the development and progression of many malignancies 25. Thus, we further evaluated the effects of metabolic syndrome components on malignancy individually and jointly. We found that when other metabolic traits were present with dyslipidemia, there was no increase in the odds of GBC. In contrast, after excluding the interaction of diabetes and obese, the effects of dyslipidemia on GBC were stronger (adjusted OR 1.915 vs 2.525). Previous studies reported that type 2 diabetes increased the risk of GBC 26. In our cohort, when interference of obesity was excluded, dyslipidemia was associated with a 2.5-fold increase in the odds of GBC, which was higher than any other metabolic syndrome components. In contrast, obesity was a protective factor for GBC. Dyslipidemia with obesity had no statistical significance (*P* = 0.631). Identifying these metabolic syndrome components in clinical practice should not be too challenging. Thus, these metabolic risk factors we identified, especially dyslipidemia, may serve as important targets for secondary prevention to modify the progression of malignancy in patients with gallbladder lesions.

Low HDL level was associated with an approximately 3-fold increase in the risk of malignancy for patients with gallbladder lesions in this study (Table 5). We also identified that low HDL was associated with a 5-fold increase in the risk of malignancy for patients with neoplastic gallbladder lesions (Supplementary Table 2). HDL is a heterogeneous mixture of macromolecules 27 and has various functions including anti-atherosclerotic, anti-oxidative, anti-inflammatory, anti-thrombotic and anti-apoptotic, as well as involving in immune modulation and endothelial protection 28. The role of HDL in cancer is still controversial. Some suggest their anti-cancer functions 29. While other studies reported the association between HDL and the risk of obesity-related cancers, and revealed an association between low HDL level and cancer risk 21. Decreased HDL was suggested as a hallmark of cancer-induced dyslipidemia 30, which was due to cancer-related inflammation and cholesterol effluxion toward the sites of cancer 31. It has also been reported that low HDL is an independent risk factor for BGP 32-34. Patients with BGP had higher TG and lower HDL levels 32. Another study revealed that high serum lipid levels played an important role in gallstone development and biliary carcinogenesis 12. The high TG and low HDL levels were closely associated with increased circulating proinflammatory cytokines, including TNF-α, IL-1, IL-6 and reactive oxygen species 12. Dyslipidemia was associated with the formation and development of gallbladder lesions and gallbladder carcinogenesis and our results also support this association.

Our data may inform intensive malignancy surveillance efforts in patients with gallbladder lesions and dyslipidemia. Based on our results, a hierarchical, stepwise and risk-stratification approach may be provided to investigate more targeted surveillance. For example, prioritising patients with gallbladder lesions and low HDL levels for more frequent follow-ups may be more effective and cost effective for early detection of GBC.

This study has some limitations. This is a retrospective analysis of enrolled patients from three hospitals in Shanghai. The generalisability of our results needs further validation. Some characteristics including tumor markers, gallbladder wall thickening, and patient lifestyles were not record in our multicenter database and not included in the analysis. The number of patients in GBC group is small, so there might be risks of bias. Although the metabolic syndrome components present targets for secondary prevention of malignancy, our data do not examine treatments. Future clinical trials of risk reduction will be able to reveal if such strategies are effective.

In conclusion, this is the first study indicates that dyslipidemia is associated with an increased risk of malignancy in patients with gallbladder lesions, regardless of the presence of hypertension, diabetes and obesity. Our findings highlight that patients with gallbladder lesions and low HDL levels may need more frequent follow-ups for early detection of GBC, and may be important target for secondary prevention.

## Supplementary Material

Supplementary tables.

## Figures and Tables

**Table 1:** Baseline characteristics of patients with gallbladder lesions

	Benign gallbladder polyps (n = 567)	Gallbladder cancer (n = 58)	P value
Gender			0.990
Male	284 (50.1)	29 (50.0)	
Female	283 (49.9)	29 (50.0)	
Age, mean (SD)	49.94 (12.73)	65.84 (9.98)	**< 0.001**
Number of polyps			**< 0.001**
Single	201 (35.4)	41 (70.7)	
Multiple	366 (64.6)	17 (29.3)	
Total bilirubin	18.40 (47.80)	44.15 (88.99)	**0.001**
Maximum diameter of polyps	6.49 (4.60)	26.26 (22.66)	**< 0.001**
With stones	118 (20.8)	2 (3.4)	**0.001**
BMI ≥ 25	202 (35.6)	13 (22.4)	**0.044**
Hypertension	275 (48.5)	34 (58.6)	0.142
Diabetes	84 (11.3)	10 (17.2)	0.181
Dyslipidemia	239 (42.2)	37 (63.8)	**0.002**
Total cholesterol ≥ 6.2	87 (15.3)	6 (10.3)	0.308
TG ≥ 2.3	72 (12.7)	9 (15.5)	0.543
Decreased HDL	144 (25.4)	31 (53.4)	**< 0.001**

TG: Triglycerides; HDL, high-density lipoprotein. BMI: body mass index.

**Table 2:** Univariate and multivariate analyses of the risk factors for gallbladder lesions

	Univariate analysis				Multivariate analysis		
	OR	95% CI	P		Adjusted OR	95% CI	P
Age	1.130	1.095-1.165	**< 0.001**				
Gender	0.996	0.580-1.711	0.990				
Number of polyps	0.228	0.126-0.411	**< 0.001**		1.307	0.566-3.020	0.531
Total bilirubin	1.005	1.001-1.008	**0.005**		1.003	0.998-1.009	0.222
Maximum diameter of polyps	1.180	1.133-1.230	**< 0.001**		1.162	1.107-1.219	**< 0.001**
Stones	0.136	0.033-0.565	**0.006**		0.178	0.036-0.882	**0.034**
Hypertension	1.504	0.870-2.602	0.144		1.116	0.478-2.609	0.799
Diabetes	1.637	0.790-3.395	0.185		0.768	0.240-2.465	0.768
Dyslipidemia	2.418	1.380-4.237	**0.002**		2.674	1.173-6.094	**0.019**
BMI ≥ 25	0.522	0.275-0.991	**0.047**		0.552	0.228-1.338	0.188

Model adjusted for age and sex. OR, odds ratio; CI, confidence interval;

**Table 3:** Types of metabolic traits of patients with gallbladder lesions

Metabolic syndrome components	Benign gallbladder polyps (567)	Gallbladder cancer (58)	P value
Hypertension	275 (48.5)	34 (58.6)	0.142
Hypertension Excluding Diabetes	230 (40.6)	29 (50.0)	0.165
Hypertension Excluding Dyslipidemia	152 (26.8)	10 (17.2)	0.113
Hypertension Excluding BMI ≥ 25	153 (27.0)	24 (41.1)	**0.020**
Diabetes	84 (11.3)	10 (17.2)	0.181
Diabetes Excluding Hypertension	19 (3.4)	5 (8.6)	**0.047**
Diabetes Excluding BMI ≥ 25	35 (6.2)	7 (12.1)	0.088
Diabetes Excluding Dyslipidemia	38 (6.7)	2 (3.4)	0.335
Dyslipidemia	239 (42.2)	37 (63.8)	**0.002**
Dyslipidemia Excluding Hypertension	116 (20.5)	13 (22.4)	0.726
Dyslipidemia Excluding Diabetes	213 (37.6)	29 (50.0)	0.064
Dyslipidemia Excluding BMI ≥ 25	147 (25.9)	29 (50.0)	**< 0.001**
Dyslipidemia and Hypertension	123 (21.7)	24 (41.4)	**0.001**
Dyslipidemia and Diabetes	26 (4.6)	8 (13.8)	**0.003**
Dyslipidemia and BMI ≥ 25	92 (16.2)	8 (13.8)	0.630
BMI ≥ 25	202 (35.6)	13 (22.4)	**0.044**

**Table 4:** Univariate and multivariate analyses of the association of types of metabolic syndrome components for gallbladder lesions

	Univariate analysis				Multivariate analysis		
	OR	95% CI	P		Adjusted OR	95% CI	P
Age	1.130	1.095-1.165	**< 0.001**				
Gender	0.996	0.580-1.711	0.990				
Hypertension	1.504	0.870-2.602	0.144		0.911	0.496-1.675	0.765
Hypertension Excluding Diabetes	1.465	0.853-2.518	0.167		1.221	0.671-2.222	0.512
Hypertension Excluding Dyslipidemia	0.569	0.281-1.153	0.117		0.438	0.201-0.953	**0.038**
Hypertension Excluding BMI ≥ 25	1.910	1.097-3.325	**0.022**		1.227	0.662-2.273	0.516
Diabetes	1.637	0.790-3.395	0.185		0.754	0.326-1.741	0.508
Diabetes Excluding Hypertension	2.721	0.977-2.721	0.056		1.821	0.559-5.929	0.320
Diabetes Excluding BMI ≥ 25	2.086	0.882-4.934	0.094		0.962	0.353-2.619	0.962
Diabetes Excluding Dyslipidemia	0.497	0.117-2.116	0.344				
Dyslipidemia	2.418	1.380-4.237	**0.002**		2.164	1.165-4.021	**0.015**
Dyslipidemia Excluding Hypertension	1.128	0.586-2.151	0.726				
Dyslipidemia Excluding Diabetes	1.162	0.967-2.858	0.066		1.915	1.039-3.526	**0.037**
Dyslipidemia Excluding BMI ≥ 25	2.857	1.652-4.924	**< 0.001**		2.525	1.365-4.669	**0.003**
Dyslipidemia and Hypertension	2.548	1.456-4.458	**0.001**		1.721	0.927-3.195	0.085
Dyslipidemia and Diabetes	3.329	1.432-7.740	**0.005**		1.406	0.544-3.635	0.482
Dyslipidemia and BMI ≥ 25	0.826	0.379-1.800	0.631				
BMI ≥ 25	0.522	0.275-0.991	**0.047**		0.528	0.262-1.067	0.075

Model adjusted for age and sex.

**Table 5:** Comparison of different indexes of dyslipidemias for gallbladder lesions

	Univariate analysis				Multivariate analysis		
	OR	95% CI	P		Adjusted OR	95% CI	P
Dyslipidemia	2.418	1.380-4.237	**0.002**		2.164	1.165-4.021	**0.015**
Total cholesterol ≥ 6.2	0.637	0.265-1.528	0.312				
TG ≥ 2.3	1.263	0.595-2.680	0.543				
Decreased HDL	3.373	1.947-5.843	**< 0.001**		3.035	1.645-5.600	**< 0.001**

Model adjusted for age and sex.

## Abbreviations

BGP: Benign gallbladder polyps
GBC: Gallbladder cancer
TG: Triglycerides
HDL: High-density lipoprotein
BMI: body mass index
OR: odds ratio
CI: confidence interval
HDL: high density lipoprotein
