# Supplementary material for: Effect of metabolic syndrome components on the risk of malignancy in patients with gallbladder lesions
Source: J Cancer. 2021 Jan 1;12(5):1531–7. doi: 10.7150/jca.54617 (PMC7847661; doi:10.7150/jca.54617)
Supplement: Supplementary file 1 — Supplementary tables. [file jcav12p1531s1.pdf]

Supplementary Table 1. Baseline characteristics of patients with gallbladder adenomatous polyps or gallbladder cancer

	Adenomatous polyps (n = 59)	Gallbladder cancer (n = 58)	P value
Gender			0.520
Male	26 (44.1)	29 (50.0)	
Female	33 (55.9)	29 (50.0)	
Age, mean (SD)	51.31 (13.84)	65.84 (9.98)	**< 0.001**
Number of polyps			0.066
Single	32 (54.2)	41 (70.7)	
Multiple	27 (45.8)	17 (29.3)	
Total bilirubin	13.15 (5.32)	44.15 (88.99)	**0.009**
Maximum diameter of polyps	9.27 (5.70)	26.26 (22.66)	**< 0.001**
With stones	15 (25.4)	2 (3.4)	**0.001**
BMI ≥ 25	19 (32.2)	13 (22.4)	0.235
Hypertension	36 (61)	34 (58.6)	0.792
Diabetes	7 (11.9)	10 (17.2)	0.409
Dyslipidemia	27 (45.8)	37 (63.8)	0.050
Total cholesterol ≥ 6.2	11 (18.6)	6 (10.3)	0.203
TG ≥ 2.3	6 (10.2)	9 (15.5)	0.387
Decreased HDL	13 (22.0)	31 (53.4)	**< 0.001**

TG: Triglycerides; HDL, high-density lipoprotein. BMI: body mass index.

Supplementary Table 2. Univariate and multivariate analyses of the risk factors between gallbladder adenomatous polyps and gallbladder cancer

	Univariate analysis			Multivariate analysis		
	OR	95% CI	P	Adjusted OR	95% CI	P
Age	1.104	1.061-1.149	**< 0.001**			
Gender	1.269	0.613-2.627	0.521			
Number of polyps	0.491	0.229-1.054	**0.068**	3.002	0.812-11.101	0.099
Total bilirubin	1.040	0.997-1.086	**0.070**	1.044	0.957-1.138	0.337
Maximum diameter of polyps	1.099	1.052-1.147	**< 0.001**	1.102	1.036-1.172	**0.002**
Stones	0.105	0.023-0.483	**0.004**	0.119	0.018-0.797	**0.028**
Hypertension	0.905	0.432-1.896	0.792			
Diabetes	1.548	0.546-4.390	0.412			
Decreased HDL	4.063	1.820-9.071	**0.001**	5.023	1.502-16.801	**0.009**
BMI ≥ 25	0.608	0.267-1.387	0.237			

Model adjusted for age and sex.

OR, odds ratio; CI, confidence interval;
